# A new tibial insert design with ball-in-socket medial conformity and posterior cruciate ligament retention closely restores native knee tibial rotation after unrestricted kinematic alignment

**DOI:** 10.1186/s40634-023-00671-3

**Published:** 2023-11-15

**Authors:** Saúl Pacheco Elorza, Ed O’Donnell, Alexander J. Nedopil, Stephen M. Howell, Maury L. Hull

**Affiliations:** 1 Department of Mechanical Engineering, University of California Davis, Davis, CA 95616 USA; 2 Department of Orthopaedic Surgery, University of California Davis Medical Center, Sacramento, CA 95817 USA; 3 Adventist Memorial Lodi Medical Center, Lodi, CA 95240 USA; 4 Department of Biomedical Engineering, University of California Davis, Davis, CA 95616 USA; 5 Department of Biomedical Engineering, Department of Mechanical Engineering, Department of Orthopaedic Surgery, University of California Davis, Davis, CA 95616 USA

**Keywords:** Medial conformity, Total knee replacement, Tibiofemoral kinematics, Lowest point method, Single-plane fluoroscopy, Flat lateral articular surface, Medial pivot, Healthy knee

## Abstract

**Purpose:**

In total knee arthroplasty (TKA) with posterior cruciate ligament (PCL) retention, the medial and lateral insert conformity that restores in vivo native (i.e., healthy) knee tibial rotation and high function without causing stiffness is unknown. The purpose was to determine whether a ball-in-socket (B-in-S) medially conforming (MC) and flat lateral insert implanted with unrestricted kinematic alignment (KA) TKA and PCL retention restores tibial rotation to native.

**Methods:**

One group of 25 patients underwent unrestricted KA TKA with manual instruments. Another group of 25 patients had native knees. Single-plane fluoroscopy imaged each knee while patients performed step-up and chair rise activities. Following 3D model-to-2D image registration, anterior–posterior (A-P) positions of the femoral condyles were determined. Changes in A-P positions with flexion were used to determine tibial rotation.

**Results:**

At maximum flexion, mean tibial rotations of KA TKA knees were comparable to native knees (Step up: 12.3° ± 4.4° vs. 13.1° ± 12.0°, *p* = 0.783; Chair Rise: 12.7° ± 6.2° vs. 12.6° ± 9.5º, *p* = 0.941). However, paths of rotation differed in that screw home motion was less evident in KA TKA knees. At 8 months follow-up, the median Forgotten Joint Score was 69 points (range 65 to 85), the median Oxford Knee Score was 43 points (range 40 to 46), and mean knee flexion was 127º ± 8°.

**Conclusions:**

The ball-in-socket medial, flat lateral insert and PCL retention implanted with unrestricted KA TKA restored in vivo native knee tibial rotation at maximum flexion for each activity and high function without stiffness. Providing high A-P stability, this implant design might benefit patients desiring to return to demanding work and recreational activities.

**Level of evidence:**

Therapeutic – Level II.

## Introduction

Because patient satisfaction after total knee arthroplasty (TKA) is strongly correlated to the ability to perform everyday activities [1], the in vivo kinematics should closely restore those of the native knee. Tibial rotation during everyday activities is an essential descriptor of knee kinematics. Moreover, restoring native internal tibial rotation during flexion provides the kinematic benefit of decreasing the Q-angle, which might reduce the risks of patellar tilt, lateral patellar displacement, and anterior knee pain [2, 3].

In unrestricted kinematic alignment (KA) TKA, restoration of native tibial rotation hinges on restoring the internal–external tibial rotation axis to native. A necessary but not sufficient requirement is that limb alignment, knee alignment, and joint lines be restored to native without ligament release. This requirement is satisfied inherently by unrestricted KA TKA where resections of the distal and posterior femoral condyles and the tibial plateau after correcting for wear preserve native alignments without ligament release [4]. A second requirement is that the design of the components and particularly the tibial insert restore the articular surfaces and soft tissue constraints of the native knee.

**Fig. 1:**
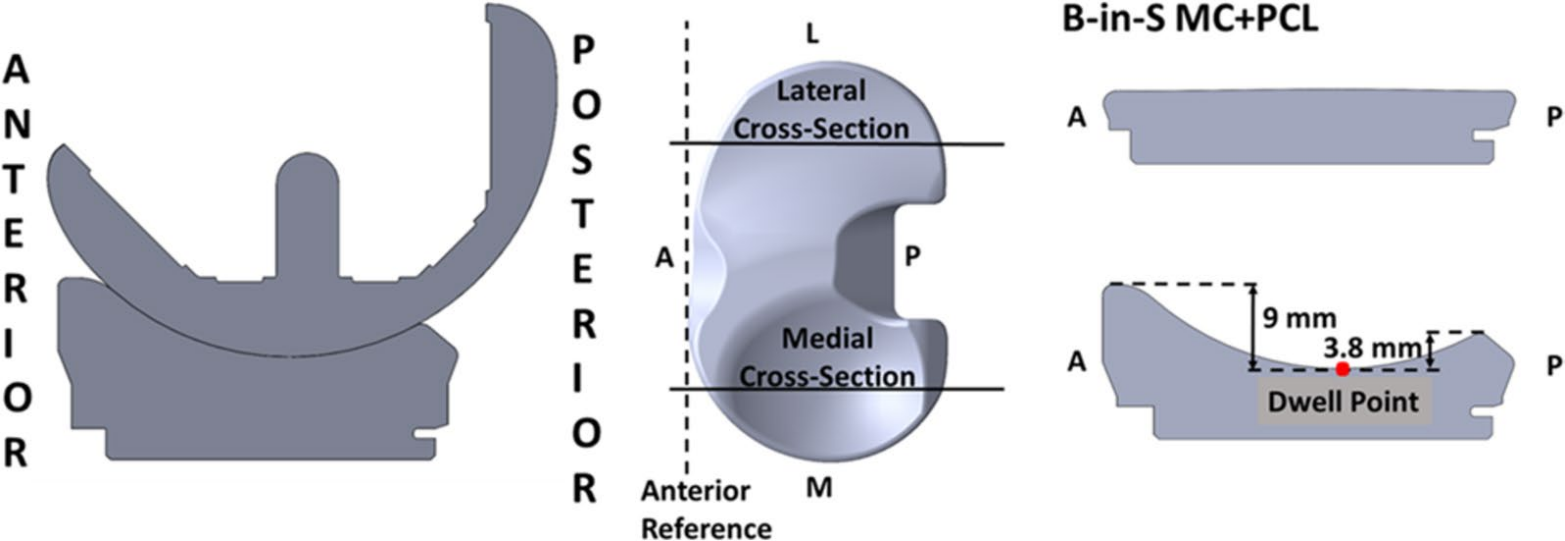
Cross-section views of the insert in the medial and lateral compartments offering ball-in-socket (B-in-S) medial conformity (MC), a flat articular surface in the lateral compartment, and PCL retention

Based on results from studies of passive motion, three design variables of a tibial insert have the potential to affect tibial rotation in weight-bearing everyday activities. One is the PCL condition; PCL resection caused a 50% loss of internal tibial rotation that was not correctable by using a thicker insert or by reducing the posterior slope of the tibial resection [5]. A second is the level of medial conformity. A ball-in-socket (B-in-S) medial conforming (MC) insert with PCL retention (+ PCL) (Fig. 1) restored 2° more internal tibial rotation relative to a less than ball-in-socket medial conforming insert during passive motion [6]. The maximum anterior–posterior (A-P) stability provided by the medial ball-in-socket forces the tibia to pivot medially thus preventing paradoxical anterior motion of the medial femoral condyle. A final design variable is the level of lateral conformity. During passive extension, an insert without anterolateral conformity enabled 4° more external tibial orientation than one with anterolateral conformity [6]. At 90° flexion, an insert without posterolateral conformity enabled 5° more internal tibial orientation than one with posterolateral conformity [6]. Hence, an insert with a flat lateral articular surface replicates the relative freedom-of-movement of the native knee’s lateral compartment [7].

As there are no reports of in vivo kinematics of a new tibial insert designed to replicate the articular surfaces and soft tissue constraints of the native knee while retaining the PCL, the purpose of the present study was to determine whether a ball-in-socket medial and flat lateral insert implanted with unrestricted KA TKA and PCL retention restores the native knee’s tibial rotation during step-up and chair rise activities. If tibial rotation was restored to native, then this insert design, which maximizes anterior–posterior (A-P) stability due to ball-in-socket medial conformity and restores native tibial rotation due to PCL retention, could be recommended particularly for those patients desiring to return to demanding work and recreational activities.

## Methods

This paper reports a secondary analysis of fluoroscopic images collected for two groups of 25 patients each (Table 1). One group included 25 native knees [8] and the other included 25 TKA knees [9] implanted with a constant radius femoral component and B-in-S MC + PCL insert (GMK Sphere, Medacta, Castel San Pietro, Switzerland) (Fig. 1) using caliper-verified unrestricted KA TKA. Retaining the PCL, the insert was a new design never before implanted in patients and tested for kinematic function. The interested reader is referred to the previous publications [8, 9] for descriptions of methods of patient recruitment and image acquisition.

**Table 1:** Comparison of patient demographics between the unrestricted KA TKA and native knee groups. Values are mean ± standard deviation (range)

Parameters	B-in-S MC + PCL (*N* = 25)	Native (*N* = 25)	Significance
Age (years)	68 ± 8 (52–81)	64 ± 7 (52–82)	*p* = 0.137
Sex (male) n	14 (56%)	14 (56%)	*p* = 1.00
BMI (kg/m^2)	31.0 ± 5.4 (22.2–42.2)	29.0 ± 4.8 (22–40)	*p* = 0.178
Time to follow-up (months)	8 ± 1 (6–9)	n.a	n.a
ACL condition (intact) n (%)	17 (68%)	n.a	n.a
Knee extension (deg)	7 ± 6 (0–22)	n.a	n.a
Knee flexion (deg)	115 ± 13 (85–138)	n.a	n.a
Varus deformity n (%)	17 (68%)	n.a	n.a
Valgus ( +)/varus (-) (deg)	3.7 ± 4.6 (-4.0 to 15.4)	n.a	n.a
Kellgren-Lawrence grade			
II	3 (13%)	n.a	n.a
III	13 (52%)	n.a	
IV	8 (32%)	n.a	
Preoperative functional outcome scores			
Oxford Knee Score (48 best, 0 worst)	29 (21–37)	n.a	n.a
Knee Society Score (100 best, 0 worst)	50 (39–70)	n.a	n.a

### Surgical technique

Using a mid-vastus approach, unrestricted caliper-verified KA TKA was performed using a previously described technique [4]. Unrestricted caliper-verified KA TKA is a patient-specific approach in which the resections are made to restore the pre-arthritic, native alignments of the limb and joint lines without ligament release [10]. Using manual instruments, the femoral component is set to within 0.3° external ± 1.1° (mean ± SD) to the native flexion–extension (F-E) plane [11]. Mean differences in the native joint lines are limited to 0.2 mm in proximal–distal and anterior–posterior (A-P) positioning and 0.2° in varus-valgus and internal–external (I-E) axial positioning [12]. Varus-valgus orientation of the tibial baseplate is set within 0.0° ± 1.8° of that of the contralateral healthy knee [4] and posterior slope is set within -0.2° ± 2.5° of the preoperative posterior slope [13]. The tibial baseplate is sized by selecting the baseplate which best fits within the cortical border of the tibial resection surface, leading to I-E positioning within 2° external ± 5° (mean ± SD) [11]. The hip-knee-ankle angle (HKAA), distal lateral femoral angle (DLFA), and proximal medial tibial angle (PMTA) are restored to within ± 3° of native with frequencies of 95%, 97%, and 97%, respectively [4]. PCL sufficiency was checked by palpation when put under tension using a laminar spreader after the resections were made, by palpation with trial components sans tibial insert in place, and by a posterior drawer test with the knee at 90º flexion.

### Fluoroscopic imaging and image processing

Briefly, patients performed two everyday activities. In the step-up activity, the subject put their foot on a 22 cm high step and lifted themselves as though they were climbing a set of stairs. In the chair rise activity, the subject began seated with their knee flexed at 90° and stood up to full extension. Fluoroscopic images were obtained at 15 frames/s using an oblique sagittal view of approximately 15° anterior.

Fluoroscopic images were selected for analysis at 60°, 45°, 30°, 15°, and 0° for step up and 90°, 60°, 30°, and 0° for chair rise. Flexion angles were measured as the included angle between the anatomic axes. Anatomic axes were the lines connecting the midpoints of the cross-sections at 7 cm and 12 cm above the joint line for the femur and 5 cm and 12 cm below the joint line for the tibia. Each image and the corresponding patient-specific 3D bone models developed from MR images of the native knee [8] or the manufacturer supplied CAD models of the TKA components were imported into open-source software for the native knee (JointTrack Manual, https://sourceforge.net/projects/jointtrack/) and for the TKA knee (JointTrack Auto, https://github.com/BRIO-lab/Joint-Track-Machine-Learning). Although each program determined the absolute position and orientation of the respective 3D models, JointTrack Auto could not be used for 3D bone models thus necessitating the use of JointTrack Manual. To improve precision in registering 3D bone models using JointTrack Manual [14], the 3D model-to-2D image registration was performed three times at each flexion angle of interest and the resultant femoral condylar A-P positions were averaged. Since the precision of JointTrack Auto using CAD models is significantly better than JointTrack Manual using 3D bone models [14], the 3D model-to-2D image registration was performed once for the CAD models.

To equivalently determine the A-P positions of the femoral condyles on the tibia for the KA TKA knee and the native knee, the tibial resection plane was simulated on the native knees. Coordinate systems were established on the plane of the simulated tibial resection to report the A-P positions of each femoral condyle. 3D models of the native femur were imported into commercial software (Geomagic Control, 3D Systems, Cary, NC). The posterior surfaces of the femoral condyles were superimposed to define the sagittal plane and the same transformation was applied to 3D models of the native tibia. The axial plane was parallel to the medial tibial articular surface and perpendicular to the sagittal plane. The plane of the simulated tibial resection was found by translating a plane parallel to the medial tibial articular surface 10 mm distally from the center of the medial tibial condyle [15]. The varus-valgus angle of the plane of the simulated tibial resection was adjusted by increments of 2° until the thicknesses of the medial and lateral tibial condyles (defined as the shortest distance between the center of the tibial condyle and the plane of simulated tibial resection in the frontal view) were within ± 0.5 mm [4]. The center of a bounding box drawn around the contour of the tibia in the simulated tibial resection defined the origin of the tibial coordinate system in the native knee. The A-P position of each femoral condyle (positive anterior) was determined by finding the lowest point with respect to the surface of the simulated tibial resection.

In the KA TKA knee, the A-P position of each femoral condyle with respect to the center of the A-P depth of the baseplate was determined. A bounding box drawn around the baseplate and the midline in the medial–lateral direction served as the reference to record the A-P positions of the medial and lateral femoral condyles (positive anterior) [8]. The A-P position of each femoral condyle in the baseplate bounding box was indicated by the A-P position of the lowest point of each femoral condyle on the plane of the tibial resection [16–18].

The A-P positions of the native and KA TKA knees were standardized to the 50 mm A-P dimension of the mid-sized tibial baseplate (Size 4, Medacta GMK Sphere). Standardization involved multiplying each patient’s A-P position by the ratio of the A-P dimension of the mid-sized baseplate to the A-P dimension of their implanted baseplate or native tibial plateau. To quantify tibial rotation., I-E rotation was the angle between medial-lateral lines connecting the non-standardized A-P positions of the medial and lateral femoral condyles at largest and smallest flexion angles in the flexion angle range of interest.

At the time of imaging, maximum extension and flexion were measured using a goniometer with the patient seated and actively extending and flexing the knee. Also, patients completed the Oxford Knee Score, the Forgotten Joint Score, the WOMAC Score, and the Oxford Knee Activity and Participation questionnaires.

### Statistical analysis

The mean and standard deviation described continuous variables including preoperative characteristics, the A–P movements of the medial and lateral femoral condyles, and the I–E rotation of the tibia on the femur. To determine whether internal tibial rotation with the B-in-S MC + PCL insert was different than that of the native knee at the limit of movement (i.e. maximum flexion for an activity), a two-sample t-test was performed for each activity. The dependent variable was the internal tibial rotation at maximum flexion referenced to the initial angle from the medial–lateral line at extension. A two-sample t-test also was performed for each 30° and 15° increment of flexion angle for chair rise and step up, respectively, to determine the arcs of extension where differences in tibial rotation occurred. An arc of extension was a 30° change in flexion angle for chair rise (e.g. 60° to 30°) and a 15° change for step up (e.g. 45° to 30°). Hence there were three and four arcs of extension for chair rise and step up, respectively. A Pearson’s chi-square test determined differences in categorical variables. The median and interquartile range described the patient-reported outcome measures (Forgotten Joint Score, Oxford Knee Score, WOMAC Score, and Oxford Activity and Participation, Table 2). Significance was set at *p* < 0.05 for all tests.

**Table 2:** Post-operative patient-reported outcome scores and knee extension and flexion for the B-in-S MC + PCL insert. Values presented are the median (interquartile-range) and mean ± standard deviation (range)

Outcomes	B-in-S MC + PCL
Oxford Knee Score (48 best, 0 worst)	43 (40–46)
Forgotten Joint Score (100 best, 0 worst)	69 (65–85)
WOMAC (0 best, 96 worst)	11 (4–20)
Oxford activity and participation (100 best, 0 worst)	91 (72–96)
Knee extension (deg)	0 ± 1 (- 2 to 1)
Knee flexion (deg)	127 ± 8 (110–144)

A power analysis confirmed that with 25 patients in each group with an α = 0.05 and (1-β) ≥ 0.80, a difference of 2.8° in tibial rotation in arcs of extension could be detected. The power analysis was performed using standard deviations of rotations in arcs of extension of 2.9° for KA TKA knees and 4.6° for native knees. Note that the power analysis was performed for tibial rotation in arcs of extension rather than tibial rotation at maximum flexion because differences in tibial rotation at maximum flexion were minimal (i.e. less than 1°) for both chair rise and step up hence not clinically important.

A previous ICC analysis determined the repeatability and reproducibility of the registration method for the native knee and revealed moderate to good agreement [8]. Another ICC analysis determined the repeatability and reproducibility of the registration method for the TKA knee and revealed good to excellent agreement [14].

## Results

**Fig. 2:**
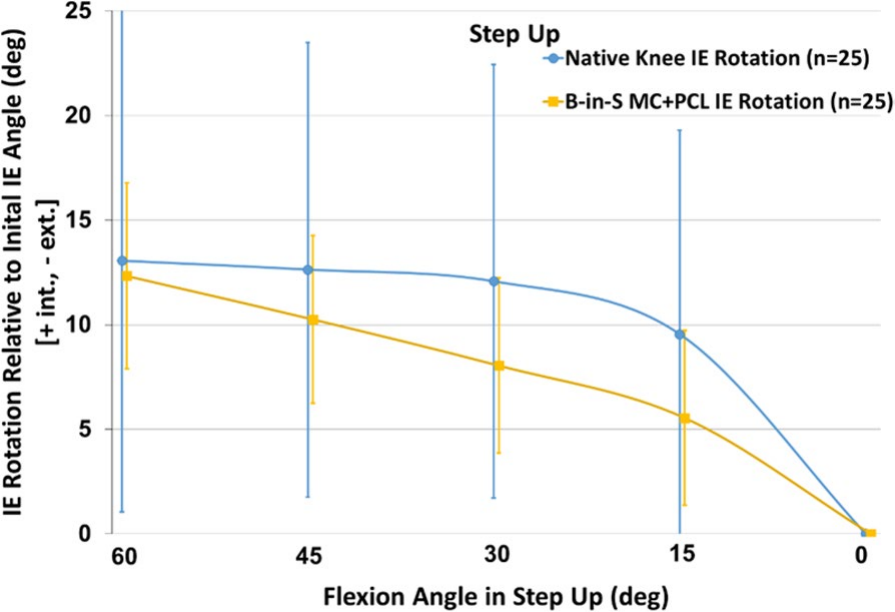
Mean tibial rotation relative to the initial angle at extension as a function of flexion angle during step up. Error bars are ± 1 standard deviation

**Fig. 3:**
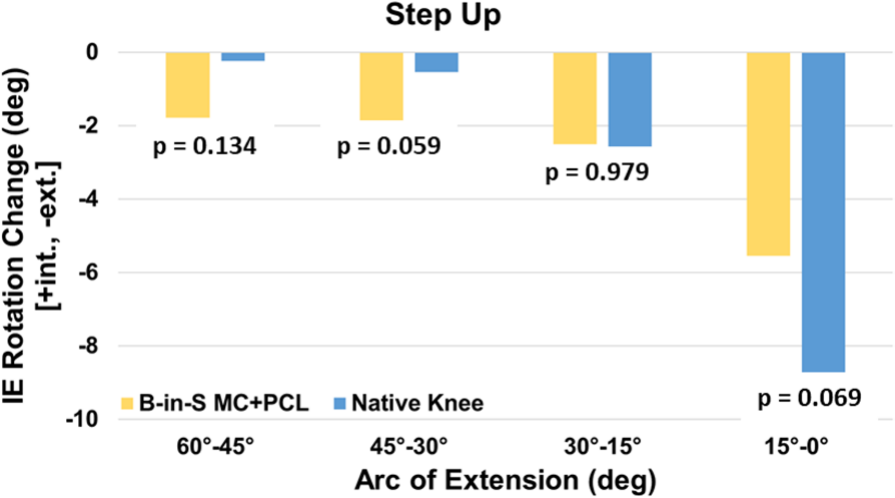
Mean change in tibial rotation in four arcs of extension during a step up

For the step up, the KA TKA and native knees exhibited comparable mean tibial rotations at maximum flexion (12.3° ± 4.4° vs 13.1° ± 12.0°, *p* = 0.783) (Fig. 2). However, the paths differed with most of the rotation occurring during the final 15° of knee extension for the native knee whereas rotation was progressive for the KA TKA knee (Fig. 3). The KA TKA knee exhibited greater rotation than the native knee from 45° to 30° and less rotation from 15° to 0° and trended towards significance (*p* = 0.059 and *p* = 0.069, respectively).

**Fig. 4:**
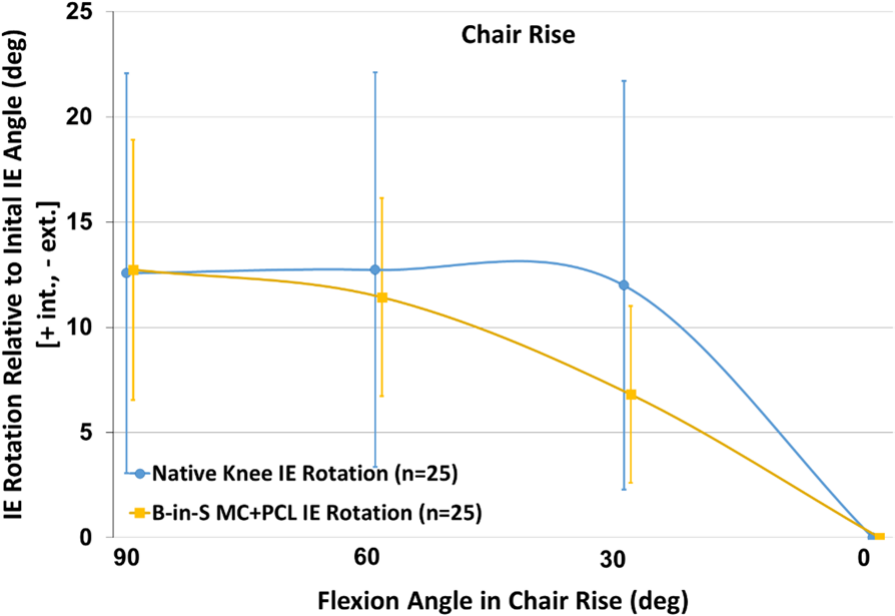
Mean tibial rotation relative to the initial angle at extension as a function of flexion angle during chair rise. Error bars are ± 1 standard deviation

**Fig. 5:**
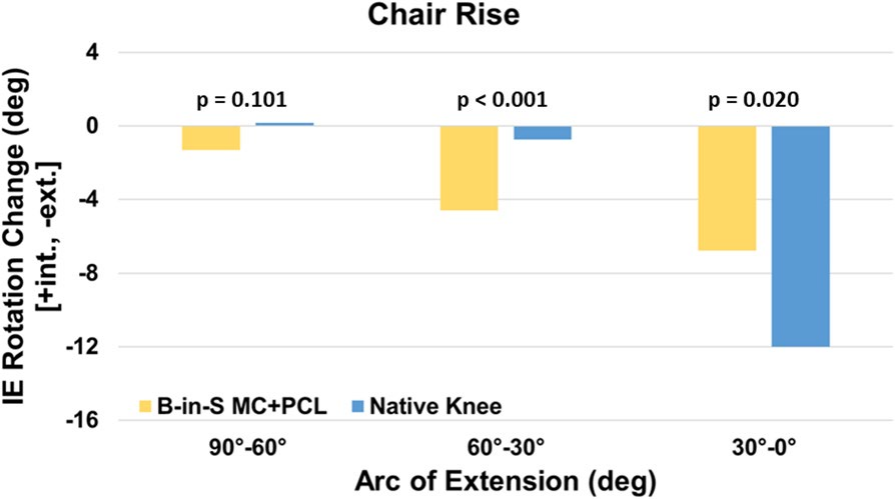
Mean change in tibial rotation in three arcs of extension during a chair rise

Likewise for the chair rise, the KA TKA and native knees exhibited comparable tibial rotation at maximum flexion (12.7° ± 6.2° vs 12.6° ± 9.5º, *p* = 0.941) (Fig. 4). However, similar to the step-up exercise, the paths differed with the native knee exhibiting most of the rotation in the final 30° of extension whereas rotation was progressive for the KA TKA knee (Fig. 5). As a result, the KA TKA knee exhibited greater rotation than the native knee from 60° to 30° (*p* < 0.001) and less rotation from 30° to 0° of flexion (*p* = 0.020).

At a mean follow-up time of 8 months, the KA TKA group exhibited a median Oxford Knee Score (OKS) of 43 (range 40 to 46), median Forgotten Joint Score of 69 (range 65 to 85), median Western Ontario and McMaster Universities Arthritis Index (WOMAC) of 11 (range 4 to 20), and median OKS-Activity and Participation Questionnaire (APQ) score of 91 (range 72 to 96) (Table 2). Knee flexion was 127° ± 8° (Table 2).

## Discussion

The most important findings were that 1) the KA TKA knee exhibited no differences in tibial rotation at maximum flexion for both activities, 2) paths of rotation differed between the KA TKA knee and the native knee, and 3) patient-reported outcome scores and flexion were all high.

There were no differences in tibial rotation at maximum flexion between the KA TKA knee and the native knee during the step-up and chair rise activities (Figs. 2 and 4). For comparison, one study reported a mean of 19° of tibial rotation during chair rise in four native knees [19]. Although this rotation is greater than the approximate 13° tibial rotation in this study, the sample size was small and the variability in tibial rotation was high (range: 2.8°-31.7°, standard deviation: 11.4°).

Although tibial rotation at maximum flexion did not differ between native and KA TKA knees, paths of rotation differed (Figs. 2, 3, 4 and 5). Most of the rotation occurred during the final 30° of extension in the native knees per the screw home mechanism whereas rotation was more progressive for the KA TKA knees. This progressive rotation in the KA TKA knees may be due to the absence of the ACL although a previous study that compared native knees to ACL-deficient knees during a deep knee bend found that ACL-deficient knees exhibited a different axial rotation path but at flexion beyond 30° [20].

Although not illustrated, a difference common to both exercises between the native knee and the KA TKA knee was a more posterior position of the medial femoral condyle of 5.1 mm for step up and 4.5 mm for chair rise at 30° of flexion than extension in the native knee but the same posterior position in the KA TKA knee. This difference occurred because of the ball-in-socket medial conformity of the insert. The ball-in-socket insert design evolved based on a previous study which showed that the medial femoral condyle has minimal A-P movement during weight-bearing knee flexion [21]. However, multiple studies have shown a more posterior position of the medial femoral condyle at 30° of flexion than extension consistent with the findings herein [8, 19, 20]. Accordingly, the ball-in-socket medial conformity might appear to over-constrain the medial femoral condyle in its ability to move posteriorly as the knee is flexed (or conversely to move anteriorly as the knee is extended). However, apparent posterior movement of the medial femoral condyle of the native knee as the knee is flexed may be a by-product of the analysis method which used lowest points on the femoral condyles to indicate A-P positions. Due to flattening of the curvature of the medial femoral condyle at extension, the lowest point moves posterior in early flexion even though the flexion facet center remains fixed relative to the tibia [21].

Though there were differences in the paths of rotation, the similarity in tibial rotation at maximum flexion during the step-up and chair rise activities is noteworthy. Everyday activities can be performed normally with a knee that flexes to 110° [22, 23]. The B-in-S MC + PCL insert with a flat lateral articular surface allowed the knee to flex well past 110° (Table 2) so that these activities could be performed with similar tibial rotation to that of the native knee albeit with a different path of rotation. It is unclear whether solely restoring the magnitude of rotation without emulating the same path of rotation as the native knee might affect patient-reported outcome scores. However, despite the difference in paths, the KA TKA knees exhibited high post-operative patient-reported outcome scores at a mean follow up of 8 months (Table 2).

A methodological issue, which has the potential to affect the results herein, concerns the reference plane used to report A-P positions of the femoral condyles. A-P positions of the native knees were determined relative to a simulated plane of tibial resection to fairly compare A-P positions to those of KA TKA knees. Although a different reference plane would yield different A-P positions, it would not affect I-E rotations. Furthermore, since a different reference plane would systematically shift A-P positions, A-P movements (i.e. differences in A-P positions between flexion angles) can still be compared meaningfully to the results of other native knee studies.

One limitation concerns the relatively short follow-up time of 8 months for the patient-reported outcome scores. However, following unrestricted KA TKA, outcome scores generally improve at 2 years [24, 25]. While it is unknown whether outcome scores will improve with time for the B-in-S MC + PCL insert tested in this study, what can be concluded is that the values herein represent lower bounds. If any change were to occur at the longer follow-up, then that change likely would result in better not worse outcome scores.

## Conclusion

The ball-in-socket medial, flat lateral insert and PCL retention implanted with unrestricted KA TKA restored in vivo native knee tibial rotation at maximum flexion for each activity and high function without stiffness. However, screw home motion was less evident in KA TKA knees. Providing high A-P stability, this implant design might benefit patients desiring to return to demanding work and recreational activities.
